# Circulating anti-mullerian hormone as predictor of ovarian response to clomiphene citrate in women with polycystic ovary syndrome

**DOI:** 10.1186/s13048-016-0214-2

**Published:** 2016-02-11

**Authors:** Wenyan Xi, Yongkang Yang, Hui Mao, Xiuhua Zhao, Ming Liu, Shengyu Fu

**Affiliations:** Department of Obstetrics and Gynaecology, The Second Affiliated Hospital of Xi’an Jiaotong University, No. 157, Xiwu Road, Xi’an City, 710004 Shaanxi Prov. China; Department of Obstetrics and Gynaecology, The Second Affiliated Hospital of Shaanxi University of Chinese Medicine, Xianyang City, 712000 Shaanxi Prov. China

**Keywords:** Anti-Müllerian hormone, Clomiphene citrate, Ovulation induction, Polycystic ovary syndrome

## Abstract

**Background:**

To investigate the impact of high circulating AMH on the outcome of CC ovulation induction in women with PCOS.

**Methods:**

This prospective cohort observational study included 81 anovulatory women with PCOS who underwent 213 cycles of CC ovarian stimulation. Serum AMH concentrations were measured on cycle day 3 before the commencement of CC in the first cycle, which were compared between responders and CC-resistant anovulation (CRA). Logistic regression analysis was applied to study the value of serum AMH for the prediction of ovarian responsiveness to CC stimulation. The receiver-operating characteristic (ROC) curve was used to evaluate the prognostic value of circulating AMH.

**Main outcome measures:**

Serum AMH levels.

**Results:**

Women who ovulated after CC therapy had a significantly lower AMH compared with the CRA (5.34 ± 1.97 vs.7.81 ± 3.49, *P* < 0.001). There was a significant gradient increase of serum AMH levels with the increasing dose of CC required to achieve ovulation (*P* < 0.05). In multivariate logistic regression analysis, AMH was an independent predictor of ovulation induction by CC in PCOS patients. ROC curve analysis showed AMH to be a useful predictor of ovulation induction by CC in PCOS patients, having 92 % specificity and 65 % sensitivity when the threshold AMH concentration was 7.77 ng/ml.

**Conclusion:**

Serum AMH may be clinically useful to predict which PCOS women are more likely to respond to CC treatment and thus to direct the selection of protocols of ovulation induction.

## Background

Polycystic ovary syndrome (PCOS) is the most common endocrine disorder in women of reproductive age, with a prevalence of approximately 5–10 %. PCOS is the major cause of anovulatory infertility [1]. The recent studies suggest that anovulation results from ovarian follicle abnormalities in PCOS patitents are 2-fold [2, 3]. First, early follicular growth is excessive, thus women with PCOS are characterized by an excessive number of small antral follicles (2- to 3-fold that of normal ovaries). Secondly, the selection of one follicle from the increased pool of selectable follicles and its further maturation to a dominant follicle does not occur. This second abnormality in the folliculogenesis is named the follicular arrest (FA) and explains the ovulatory disorder of PCOS. Although the FA has not received yet a clear and unanimous explanation, Anti-Müllerian hormone (AMH) is considered as important contributors to this abnormality [4, 5].

AMH is producted specifically by granulose cells of early developing pre-antral and small antral follicles in the ovary. Serum AMH levels in women with PCOS are 2- to 3-fold higher than in ovulatory women with normal ovaries [6, 7], which corresponds to the 2- to 3-fold increase in the number of small follicles seen in PCOS. The increased AMH has been hypothesized may reduce follicle sensitivity to FSH and oestradiol production, thus preventing follicle selection, resulting in follicle arrest at the small antral phase with the failure of dominance.

At present, the treatment of oligo- or anovulatory infertility is referred to as induction of ovulation. Clomiphene citrate (CC) is the treatment of first choice for ovulation induction in anovulatory women with PCOS. There are 20–25 % of women, however, remain anovulatory after receiving CC medication [8] and the exact cause of CC failure in some patients remain uncertain. Indentifying factors that determine the response of women with PCOS to CC will help selecting patients who are likely to benefit from this treatment, thus avoiding fruitless treatment and improving success rates.

Recently, AMH has been characterized as a promising novel clinical marker of ovarian reserve and predicting ovarian response to gonadotrophins during in vitro fertilization (IVF) in women without PCOS [9–11]. In PCOS women, we recent found AMH levels on day 3 of the IVF stimulation cycle still positively predict ovarian response to gonadotrophins [12]. However, different from our study, the predictive meaning of AMH was considered different between women with and without PCOS, for the authors found circulating AMH levels were negatively correlated with ovarian response to gonadotrophins during ovary induction in PCOS women [13]. Hence, the results of hitherto published studies are seemed not entirely in consensus. So we designed a study to investigate whether serum AMH has a role in predicting ovary response to CC treatment in a large cohort of infertile women with PCOS.

## Methods

### Patients

Subjects included 81 anovulatory women with PCOS who were referred to our department for ovulation induction between February 2012 and June 2014. The diagnosis of PCOS was based on the Rotterdam criteria, in which at least two of the following three criteria were met: oligomenorrhea or amenorrhea, hyperandrogenaemia, and sonographic appearance of polycystic ovaries [14]. Oligomenorrhoea was defined as cycles lasting longer than 35 days. Amenorrhea was defined as cycles lasting longer than 6 months. Hyperandrogenism was diagnosed either clinically (acne/hirsutism) and/or biochemically (testosterone >0.7 ng/ml). The ovary was considered polycystic on ultrasound scan if it contained ≥12 follicles (2–9 mm in diameter) and/or measured >10 ml in volume. All patients presented with anovulatory cycles for at least 2 years. The inclusion criteria included: patients 35 years old or younger, BMI ≤30 kg/m without previous ovulation induction and partners with normal semen parameters. No PCOS patient had evidence of hyperprolactinemia, Cushing’s syndrome, congenital adrenal hyperplasia or androgen-secreting tumors.

### Ethical approval

This study was approved by the Ethics Committee of The Second Affiliated Hospital of Xi‟an Jiaotong University. All participants provided their informed consent before their involvement in this study.

### Clomiphene citrate treatment

All women received an initial dose of 50 mg/d CC from cycle d3 until d7 after spontaneous or progestagen-induced withdrawal bleeding. In the case of an absent ovarian response, daily dosage was increased to 100 mg in the following cycles. If ovulation occurred, the dose remained unaltered during subsequent cycles. First ovulation was used as the end point. The duration of all patients included in the study was at least three treatment cycles. Ovulation was assessed by midluteal serum progesterone measurement (levels >10 ng/ml indicating ovulation) combined with transvaginal sonographic monitoring of follicle growth until the appearance of a preovulatory follicle (mean diameter ≥18 mm) and subsequent follicle rupture. Responders were defined as patients who ovulated during CC therapy, independent of the dose administered. Failure to ovulate in three CC cycles despite stimulation with the maximum dose (100 mg/d) was referred to as CC-resistant anovulation (CRA). Clinical pregnancy was defined as the presence of a gestational sac with cardiacactivity as detected by transvaginal ultrasound after 35 days of ovulation.

### Hormone assays

Blood samples were collected on cycle day 3 before the commencement of CC in the first cycle of treatment to measure baseline serum concentrations of AMH. AMH was measured by using a second-generation enzyme-linked immunosorbent assay (ELISA) (Immunotech Beckman Coulter Laboratories, Villepinte, France). The analytical sensitivity of this assay is 0.14 ng/mL. Intra- and inter-assay coefficients of variation were ≤12.3 and ≤14.2 %, respectively.

Serum other hormonal concentrations including luteinizing hormone (LH), follicle stimulating hormone (FSH), testosterone (T), insulin and progesterone were measured using electrochemiluminescence immunoassay (Roche Diagnostics GmbH, Mannheim, Germany). Insulin resistance, defined by the homeostasis model assessment insulin resistance index (HOMA-IR), was calculated using the following equation: HOMA-IR = fasting insulin (IU/ml) × fasting glucose (mmol/L)/22.5 [15].

### Transvaginal scan

In the same morning of the blood tests, a transvaginal ultrasound scan was performed to assess the ovarian volume (milliliters), and antral follicles count (AFC). The volume of each ovary was calculated by measuring the ovarian diameters (D) in three perpendicular directions and applying the formula for an ellipsoid: D1 × D2 × D3 × 0.5236. For the determination of the AFC, we calculated small follicles with a diameter between 2 and 9 mm, following the recommendations as described previously [16].

### Statistical analysis

The Statistical Package for Social Sciences (SPSS 17.0, Chicago) was used for statistical analysis. Differences between responders and nonresponders were tested using the *t*-test, nonparametric test (Mann–Whitney U) and *χ*2-test as appropriate. Spearman’s correlation co-efficient was calculated to evaluate the relation of AMH to other characteristics of PCOS. Using the results of the ROC analysis, we defined an appropriate threshold level for AMH and determined the sensitivity and specificity of that threshold. Logistic regression analysis was applied to study the value of serum AMH and other study variables for the prediction of ovarian responsiveness to CC stimulation. *P* < 0.05 was considered statistically significant. Multiple logistic regression analysis with forward selection of parameters was applied with *P* < 0.10 for entry.

## Results

**Fig. 1:**
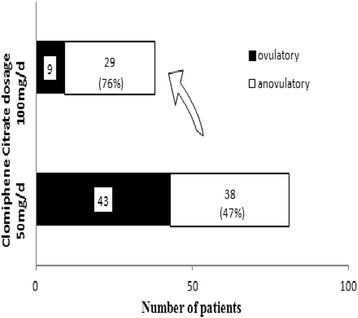
Distribution of women who do or do not ovulate after CC induction of ovulation in incremental daily doses of 50, or 100 mg for 5 subsequent days. A total of 29 women (35.8 % of the overall study group) remain anovulatory

The study included 81 anovulatory women with PCOS who received 213 cycles of CC ovulation induction. Patient characteristics are shown in Table 1. Of the 81 women included in the study, 43 (53.1 %) ovulated during ovulation induction with CC 50 mg/d. This number increased to 52 (64.2 %) after increasing CC dose up to the100 mg/d, 29(35.8 %) remaining anovulatory were considered CRA (Fig. 1). A total of 26(32.1 %) women conceived during up to three cycles of CC treatment. Of the 213 CC cycles, ovulation occurred in 114 cycles (53.5 %) and pregnancy in 26 cycles (12.2 %).

**Table 1:** Baseline characteristics of 81 anovulatory women with PCOS who received CC ovulation induction, and separated for women who do (responders) or do not ovulate (CRA) after CC induction of ovulation

Variable	All participants	CC responders	CRA	*P* value
	*n* = 81	*n* = 52	*n* = 29	
Age (years)	26.62 ± 2.53	26.98 ± 2.48	25.97 ± 2.53	NS
BMI (kg/m2)	23.79 ± 2.78	23.53 ± 2.81	24.24 ± 2.71	NS
LH(IU/L)	8.29 ± 2.37	8.01 ± 2.29	8.77 ± 2.49	NS
FSH(IU/L)	5.73 ± 1.19	5.79 ± 1.28	5.62 ± 1.03	NS
LH/FSH	1.52 ± 0.59	1.46 ± 0.58	1.63 ± 0.62	NS
T(ng/ml)	0.56 ± 0.25	0.55 ± 0.23	0.58 ± 0.29	NS
HOMA-IR	3.18 ± 1.92	3.11 ± 2.16	3.31 ± 1.41	NS
Ovarian volume (ml)	10.43 ± 1.58	9.7 ± 1.32	10.49 ± 1.74	<0.05
AFC (n)	16.11 ± 3.71	15.44 ± 3.17	17.31 ± 4.33	<0.05
AMH	6.22 ± 2.8	5.34 ± 1.97	7.81 ± 3.49	<0.001

Note: Values are mean ± SD unless otherwise indicated

*CRA* CC-resistant anovulation, *BMI* body mass index, *LH* luteinizing hormone, *FSH* follicle stimulating hormone, *T* testosterone, *HOMA-IR* the homeostasis model assessment insulin resistance index, *AFC* antral follicles count, *AMH* antimüllerian hormone, *NS* Not statistially significant

Women were divided into two groups based on their response to clomiphene citrate treatment: CC responders (*n* = 52) and CRA (*n* = 29). Patients who ovulated had a significantly lower serum AMH concentration compared with nonresponders (5.34 ± 1.97 vs.7.81 ± 3.49, *P* < 0.001). AFC and ovarian volume from responders group were statistically significantly lower than from the CRA group (*P* < 0.05). There were no significant differences between the groups in mean age, BMI, FSH, LH, LH/FSH, T and HOMA-IR (Table 1). In addition, patients who conceived had a significantly lower serum AMH concentrations compared with that of those who did not conceive (4.81 ± 2.06 vs. 6.89 ± 2.95 ng/ml, *P* < 0.01) (Table 2). When CC-resistant patients were excluded from analysis of pregnancy, serum AMH concentrations were comparable in women achieving pregnancy (*n* = 26) and those not conceiving (*n* = 26) (4.81 ± 2.06 ng/ml vs 5.67 ± 1.76 ng/ml, *P* > 0.05).

**Table 2:** Comparison the characteristics of PCOS women who conceived on CC treatment (*n* = 26) and those who did not conceive (*n* = 55)

	Pregnant	Nonpregnant	*P* value
	*n* = 26	*n* = 55	
Age (years)	27.08 ± 2.23	26.40 ± 2.65	NS
BMI (kg/m2)	23.46 ± 2.83	23.94 ± 2.77	NS
LH(IU/L)	8.05 ± 2.12	8.40 ± 2.50	NS
FSH(IU/L)	5.55 ± 1.3	5.82 ± 1.14	NS
LH/FSH	1.55 ± 0.65	1.51 ± 0.57	NS
T(ng/ml)	0.55 ± 0.22	0.58 ± 0.27	NS
HOMA-IR	3.02 ± 1.35	3.27 ± 2.69	NS
Ovarian volume (ml)	9.52 ± 1.28	10.27 ± 1.48	<0.05
AFC (n)	15.04 ± 2.82	16.62 ± 3.98	NS
AMH	4.81 ± 2.06	6.89 ± 2.95	<0.01

Spearman’s correlations between serum AMH concentrations and other characteristics of PCOS showed AMH significantly correlated with serum LH (*r* = 0.253, *P* < 0.05), ovarian volume (*r* = 0.297, *P* < 0.01) and AFC (*r* = 0.296, *P* < 0.01). No statistically significant correlation between serum AMH and BMI, FSH, LH/FSH, T and HOMA-IR could be found (Table 3). Univariate logistic regression analysis showed that AMH, AFC and ovarian volume were significant predictors of ovarian response to CC stimulation. For the multivariate logistic regression analysis using stepwise forward selection on all variables, AMH was selected in the final model, while mean ovarian volume and AFC were not (date were not shown).

**Table 3:** Spearman’s correlations between plasma AMH and other factors in women with PCOS

Variable	r	*P* value
Age (years)	−0.012	NS
BMI (kg/m2)	0.027	NS
LH (IU/L)	0.253	<0.05
FSH(IU/L)	−0.06	NS
LH/FSH	0.207	NS
T(ng/ml)	0.065	NS
HOMA-IR	0.016	NS
Ovarian volume (ml)	0.297	<0.01
AFC (n)	0.296	<0.01

**Fig. 2:**
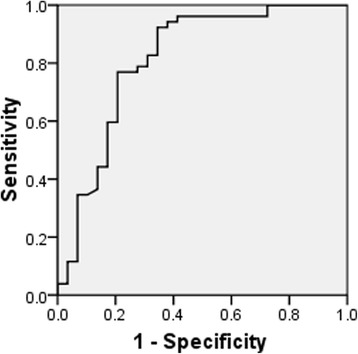
Receiver operating characteristic curve of AMH to predict clomiphene citrate resistance in patients with polycystic ovary syndrome

Figure 2 presents ROC for the sensitivity and specificity of the AMH at different levels in predicting no ovulation after CC therapy. The AMH shows a ROCAUC of 0.813 for no ovulation, indicating a useful potential for predicting CRA. Considering a serum AMH concentration of 7.77 ng/ml as cut-off, the sensitivity and specificity of predicting no ovulation were 92 and 65 % respectively. With this cut-off (7.77 ng/ml), the outcomes of CC ovarian stimulation were compared between cycles with high AMH vs. low AMH levels. Patients with AMH levels less than 7.77 ng/ml had significantly higher ovulation and pregnancy rates than those with AMH of 7.77 ng/ml or greater. In addition, patients with high AMH levels had significantly higher LH, ovarian volume and AFC (Table 4).

**Table 4:** Comparison of PCOS women with high vs low AMH using a cutoff value of 7.77 ng/ml

	AMH <7.77 ng/ml	AMH ≥7.77 ng/ml	*P* value	PPV	NPV
	*n* = 59	*n* = 22			
Age (years)	26.68 ± 2.31	26.45 ± 3.08	NS		
BMI (kg/m2)	23.54 ± 2.80	24.45 ± 2.67	NS		
LH(IU/L)	7.92 ± 2.26	9.28 ± 2.43	<0.05		
FSH(IU/L)	5.73 ± 1.16	5.73 ± 1.29	NS		
LH/FSH	1.45 ± 0.55	1.72 ± 0.66	NS		
T(ng/ml)	0.56 ± 0.22	0.56 ± 0.33	NS		
HOMA-IR	3.12 ± 2.09	3.24 ± 1.39	NS		
Ovarian volume (ml)	9.72 ± 1.31	10.69 ± 1.82	<0.01		
AFC (n)	15.24 ± 3.06	18.45 ± 4.31	<0.01		
Ovulation/patient	46/81(57 %)	6/81(7 %)	<0.001	88.5	55.2
Pregnancy/patient	23/81(28 %)	3/81(4 %)	<0.01	88.4	34.5

### AMH and dose of CC

The mean serum concentration of AMH was compared between PCOS patients who responded to CC 50 mg (*n* = 43) vs those who responded to the higher dose 100 mg (*n* = 9). The results showed a significant (*P* < 0.05) increase of serum AMH level with the increasing dose of CC (Table 5).

**Table 5:** Serum AMH concentrations in PCOS patients achieving ovulation on different doses of CC

Dose (mg)	Clinical outcome		AMH levels achieving ovulation	*P* value
	Not ovulated, n (%)	Ovulated, n (%)		
50	38 (46.9)	43 (53.1)	5.07 ± 1.8	---
100	29 (76.3)	9 (23.7)	6.64 ± 2.34	<0.05*

Note: *The mean AMH levels was compared between PCOS patients who responded to CC 50 mg vs those who responded to the dose 100 mg

## Discussion

Since the increased AMH would impair the action of FSH and contribute to the FA of PCOS, this evidence has led us to hypothesise that there is a subgroup of women with PCOS who have the higher levels of AMH and who are the more resistant to CC treatment. In this study, we really proved that patients with high AMH level are less likely to respond to CC treatment. Furthermore, we have identified a cut-off level of AMH (7.77 ng/ml), above which the chances of ovulation seem to be significantly reduced. These observations suggest that high AMH values reflect more impaired disruption in folliculogenesis and granulosa cell function.

However, it may seem paradoxical that serum AMH concentrations are known to positively predict ovarian response to gonadotrophin stimulation during IVF. For women with high AMH levels are considered to predict excessive ovarian response to gonadotropin. Meanwhile, low AMH levels indicative of a diminished ovarian reserve, is associated with poor response [17, 18]. Amer SA et al. [13] explained the contradiction may be due to the different spectrum of circulating AMH in women with and without PCOS. Since AMH levels were significantly increased in women with PCOS, they considered levels above the optimum AMH values are associated with poor ovarian response to stimulation. It is interesting to note that, in contrast to Amer SA’s opinion, Kaya et al. [19] and our previous study [12] found a positive association between serum AMH levels and ovarian responsiveness to gonadotrophins during IVF in women with PCOS. In that study as serum AMH levels increased, an increase in estrodiol levels on the day of hCG administration and the number of retrieved oocytes were observed, while the total dose of the gonadotrophins was significantly decreased. Thus, we suppose the predictive role of AMH is different in ovarian responsiveness to ovulation induction with CC and ovarian hyperstimulation with gonadotrophins for IVF treatment, because the goal of stimulation in women with anovulation is different than that in women undergoing IVF.

It is sopposed that in anovulatory women with PCOS, increasing the serum FSH level may reduce the AMH excess, thus relieving its inhibition on the follicular growth, and allowing the emergence of a dominant follicle [20]. In ovulation induction the aim should be to achieve the ovulation of a single follicle, CC thus constitutes the first line treatment of choice in PCOS women. Chronic low-dose gonadotrophins (with a starting dose 37.5 or 50U daily) have been used to stimulate ovulation in women who fail to ovulate with CC. However, both CC and low-dose gonadotrophins make the serum FSH levels increased gently and may be not enough to reduce intra-ovarian AMH to a level consistent with resumption of ovulation in women with high AMH level. Therefore, as expected the patient with higher AMH were more deeply inhibited and more likely to remain anovulatory after ovulation induction. The aim of IVF treatment, however, is normally designed to promote multifollicular development and as such will usually employ higher doses of FSH (with a starting dose at least 112.5U daily) than those used for ovulation induction. When the ‘threshold’ level of FSH for follicular growth is quickly exceeded and follicle arrest from AMH inhibition was relieved, resulting in an early visualization of multiple dominant follicles development.

Our findings are consistent with previous study by Mahran A and co-workers [21] who have evaluated the impact of circulating AMH on the success rates of CC ovulation induction in 60 women with anovulatory PCOS receiving 187 cycles of treatment, and found circulating AMH levels to be negatively correlated with the chances of ovulation. Simially, Amer SA et al. [13] have evaluated the impact of circulating AMH on the outcome of ovarian stimulation in 20 women with anovulatory PCOS undergoing 34 cycles of gonadotrophin treatment. They found circulating AMH levels to be negatively correlated with ovarian response to human menopausal gonadotrophin. On the other hand, our findings concur with those of El-Halawaty et al. [22], in that AMH levels were significantly higher in responders to CC therapy when compared to non-responder. However, their findings included a subgroup of obese PCOS women receiving a high dose of CC (150 mg/d). Only 25 % of participants in that study ovulated in response to the high CC in first cycle, which is much lower than the majority of publications reporting ovulation rates of 75–80 % after CC treatment [8].

AMH was reported to be one of the local inhibitors of FSH action by decreasing granulosa cell sensitivity to FSH [23, 24], since the antral follicles from AMH knockout mice are more sensitive to FSH than those from the wild type [25]. This effect of AMH was mainly the result of inhibited aromatase activity in granulosa cell. In keeping with this study, an inhibitory effect of AMH on FSH- induced aromatase mRNA expression and estradiol production has been shown in human GLCs [26]. Similarly, the inverse relationship between AMH and estradiol has also been found in PCOS women [6]. The fact that AMH is inhibitory to factors required for follicle growth and subsequently selection process of the dominant follicle [3], thus it is not surprising that AMH is a negatively predictive factor for ovarian response to CC therapy in PCOS women.

It is of note that AMH, LH, AFC and ovarian volume are closely related. Furthermore, AFC and ovarian volume were significantly higher in the CRA group compared with responder. These may therefore be confounding factors that could have an influence on responsiveness to CC. So we have used multiple logistic regression analysis to determine which of these factors is an independent predictor of ovulation. The analysis has shown that AMH serum level is the best overall predictor of ovarian response to CC treatment.

In current study, the AMH levels were significantly higher in non-pregnancy compared with pregnancy group. However, this difference was disappeared when CC-resistant women were excluded from the analyses. This may be due to the fact that most CC resistant patients in this study had relatively higher AMH were excluded from the non-pregnancy group.

In the present study, we found serum AMH levels with a threshold of 7.77 ng/ml had a sensitivity of 92 % and specificity of 65 % in predicting ovarian response to CC. This cut-off is greater than two times those of previously reported by Mahran A et al. [21] who reported that 3.4 ng/ml was an optimal cut-off for the prediction of CRA among 60 women with PCOS. It is possible that different kits for detecting AMH might result in substantial variation in the serum level of AMH. In addition, variations in PCOS manifestations and AMH across different racial/ethnic backgrounds may be ascribed to these differences. Therefore, it should be noted that our cutoff AMH level applies only to the AMH kit used in this study. More studies are needed to test which value would be most useful in clinical practice.

The main strength of this study is its prospective design with inclusion of anovulatory patients fulfilling the study inclusion. However, our study has certain limitations that should be noted. Definitions vary the dose required to define CC-resistance ranging from 100 mg to150 mg of CC [27, 28]. In the present study, we defined CC-resistance as failure to ovulate in three CC cycles with the maximum dose 100 mg of CC. The CC non-responder in our study may ovulate in response to 150 mg of CC administration. However, the doses in excess of 100 mg per day are not approved by Food and Drug Administration of United States. Therefore, we did not prescribe more than 100 mg per day of CC in this study.

## Conclusions

In summary, this study demonstrates that the plasma AMH can predict ovarian response to CC treatment. Therefore, measurement of serum AMH concentration for anovulatory women with PCOS before treatment may be a useful tool in predicting the outcome of CC administration. This could help with counselling PCOS patients concerning the expected success of CC treatment and may render the ovulation-induction protocols more patient-tailored and more cost-effective.

## Abbreviations

AFC: antral follicles count
AMH: Anti-Müllerian hormone
BMI: body mass index
CC: clomiphene citrate
CRA: CC-resistant anovulation
FA: follicular arrest
FSH: Follicle stimulating hormone
hCG: Human chorionic gonadotrophin
HOMA-IR: homeostasis model assessment insulin resistance index
IVF: In-vitro fertilisation
LH: Luteinizing hormone
PCOS: Polycystic ovarian syndrome
ROC: Receiver operating characteristic
T: testosterone
